# PSD-YOLO: An Enhanced Real-Time Framework for Robust Worker Detection in Complex Offshore Oil Platform Environments

**DOI:** 10.3390/s25206264

**Published:** 2025-10-10

**Authors:** Yikun Qin, Jiawen Dong, Wei Li, Linxin Zhang, Ke Feng, Zijia Wang

**Affiliations:** 1 Southampton Ocean Engineering Joint Institute, Harbin Engineering University, Harbin 150001, China; yq2c22@soton.ac.uk (Y.Q.); jd1c22@soton.ac.uk (J.D.); lz2c22@soton.ac.uk (L.Z.); kf1c22@soton.ac.uk (K.F.); zw4c22@soton.ac.uk (Z.W.); 2 College of Computer Science and Technology, Harbin Engineering University, Harbin 150001, China

**Keywords:** offshore platforms, personnel detection, YOLOv10, attention mechanism

## Abstract

To address the safety challenges for personnel in the complex and hazardous environments of offshore drilling platforms, this paper introduces the Platform Safety Detection YOLO (PSD-YOLO), an enhanced, real-time object detection framework based on YOLOv10s. The framework integrates several key innovations to improve detection robustness: first, the Channel Attention-Aware (CAA) mechanism is incorporated into the backbone network to effectively suppress complex background noise interference; second, a novel C2fCIB_Conv2Former module is designed in the neck to strengthen multi-scale feature fusion for small and occluded targets; finally, the Soft-NMS algorithm is employed in place of traditional NMS to significantly reduce missed detections in dense scenes. Experimental results on a custom offshore platform personnel dataset show that PSD-YOLO achieves a mean Average Precision (mAP@0.5) of 82.5% at an inference speed of 232.56 FPS. The efficient and accurate detection framework proposed in this study provides reliable technical support for automated safety monitoring systems, holds significant practical implications for reducing accident rates and safeguarding personnel by enabling real-time warnings of hazardous situations, fills a critical gap in intelligent sensor monitoring for offshore platforms and makes a significant contribution to advancing their safety monitoring systems.

## 1. Introduction

Offshore drilling platforms are integral to the global energy supply chain and economic development [1]. However, they operate in highly complex and hazardous environments, and the remote location of these platforms and limited escape options significantly exacerbate the severity of accidents [2]. Therefore, establishing a robust safety monitoring system for offshore platforms is essential to ensure personnel safety and mitigate accident risks [3].

In recent years, various safety monitoring systems have been proposed to ensure the safety of extraction operations. Ye et al. [4] developed a comprehensive monitoring system architecture, which includes sensor networks, data transmission, and management and evaluation subsystems, aimed at improving the safety of offshore platform structures. Xia et al. [5] introduced a monocular vision-based safety monitoring framework, implementing the grounded SAM algorithm to achieve object detection, instance segmentation and distance estimation, thus, enabling real-time early warning for offshore infrastructure.

In the above-mentioned safety monitoring system, the accurate detection of workers is crucial for subsequent tasks, such as behavior analysis and emergency response. Object detection methods based on computer vision have emerged as a key research focus. Earlier approaches relied mainly on traditional image processing and machine learning techniques to detect workers’ safety equipment [6,7,8].

However, these methods exhibit limited adaptability and are typically effective only in environments with minimal background interference and relatively simple settings. With the advancement of deep learning, neural network-based worker detection techniques have progressively become the mainstream approach, significantly enhancing detection accuracy and adaptability [9,10,11,12]. These methods have improved both the speed and accuracy of detection and have gradually been applied to the field of oil worker safety monitoring. However, the accurate detection of workers in complex environments such as offshore drilling platforms faces significant challenges, mainly reflected in the following four aspects: (1) Dynamic background noise: The dynamic changes of the sea wave background affect image quality, easily causing target features to become obscured, which in turn leads to missed detections and localization errors. (2) Structural complexity: The platform’s interior, featuring a dense network of intersecting steel frames, pipelines, and equipment [13], creates a visually cluttered background. This causes the features of workers, such as their silhouettes and clothing, to blend easily with the industrial environment, thereby increasing the risk of false detections (3) Occlusion scenarios: Due to the narrow workspace on the platform, workers are frequently and severely occluded by equipment, pipelines, or other colleagues. Such occlusion scenarios pose a significant challenge for traditional object detection methods, which often fail to identify partially visible targets, leading to a high rate of missed detections [14]. (4) Environmental uncertainty: The offshore environment is highly variable; unstable lighting conditions and adverse weather, such as heavy fog, can significantly degrade image quality, thereby compromising the reliability of the monitoring system [15].

Collectively, these complex environmental factors expose the inherent limitations of a standard architecture of a basic YOLO method. First, when faced with visual clutter from the platform’s structure and sea waves, its standard feature extraction network struggles to separate valid worker features from high-frequency background noise. Second, in scenarios with severe worker occlusion, the traditional Non-Maximum Suppression (NMS) algorithm employed by basic YOLO has a critical flaw: it aggressively discards overlapping boxes entirely rather than gradually decaying their scores, which often leads to the erroneous suppression of and missed detections for partially visible targets. Finally, under low-visibility conditions like poor lighting or heavy fog, the texture information of worker targets becomes unclear, and the basic model lacks specialized feature enhancement mechanisms for such “weak targets,” causing a significant drop in its detection robustness.

To address the challenges of worker safety detection on offshore drilling platforms, researchers have proposed various solutions. For instance, Ji et al. [16] developed a personal protective equipment (PPE) detection framework based on an improved YOLOv4. However, its performance remains limited in scenarios involving target occlusion and complex backgrounds. Gong et al. [17] introduced a deep learning approach to enhance worker action detection, but this method lacks optimization for real-time object detection and small target detection. Subsequently, they [18] proposed another detection method combining an improved YOLOv3 with transfer learning; while achieving high accuracy, the model’s multi-scale feature fusion for occluded targets still had room for improvement. Additionally, Yang et al. [19] explored a low-cost personnel localization method using the YOLO algorithm, but their study was confined to spatial localization and did not address more complex tasks like PPE detection or behavior detection. Li et al. [20] proposed the ACD-Net model to detect and recognize abnormal crew behavior in complex scenarios in real time. Although ACD-Net achieves high accuracy and low processing time, its detection accuracy and generalization ability still need improvement in harsh conditions like fog and low light, and for detecting occluded crew members.

To address the problems of worker detection in the complex environment of offshore oil platforms discussed in the aforementioned studies, this paper proposes an efficient worker detection model, the Platform Safety Detection YOLO (PSD-YOLO) algorithm, and constructs a dataset for offshore oil worker detection covering various typical operational scenarios. Experimental results demonstrate that the PSD-YOLO algorithm effectively improves the accuracy and stability of worker detection in offshore operational environments by addressing issues such as low detection accuracy for small targets in complex backgrounds and target occlusion. The main contributions of this paper are as follows:

(1) This paper proposes PSD-YOLO, a framework based on YOLOv10s specifically optimized for worker detection on offshore oil platforms. The architecture is engineered to achieve a superior balance between high accuracy and real-time performance within a lightweight structure. Consequently, the model facilitates rapid and precise worker detection in complex offshore environments, directly addressing the critical challenges inherent to this application.
(2) To enhance long-range contextual modeling, the Channel Attention-Aware (CAA) module is integrated after the 2fIB block in the backbone network. By dynamically adjusting the importance of target features, it effectively improves the focus on the target, reduces background noise interference, and further optimizes target detection accuracy in complex background environments. In the neck network, the newly designed C2fCIB_Conv2Former module replaces the original two C2f modules. Utilizing large-kernel depth-wise convolution, strengthens multi-scale feature fusion, optimizes feature integration and modeling, and improves the model’s detection ability for small targets.
(3) To address the challenge of missed detections in occlusion scenarios on offshore platforms, we incorporate the Soft Non-Maximum Suppression (Soft-NMS) algorithm. Unlike the traditional NMS, Soft-NMS gradually reduces the scores of overlapping boxes instead of suppressing them directly, significantly mitigating missed detections and thereby enhancing detection accuracy and robustness.
(4) To achieve robust adaptation in offshore environments, a strategy of Domain Adaptation via Cross-Domain Knowledge Transfer is employed. This approach enhances the model’s robustness in the unique and complex environment of offshore platforms by leveraging foundational knowledge from pre-trained models.

## 2. Related Work

With the development of deep learning technology, computer vision-based object detection has become a key technology in the field of industrial safety monitoring, playing a crucial role, especially in ensuring the safety of personnel working in high-risk environments such as offshore oil platforms. The accurate and real-time detection of workers is the foundation for implementing advanced intelligent monitoring functions such as behavior analysis, violation alerts, and emergency response. Currently, research on personnel detection in industrial scenarios can be primarily divided into two main technical approaches: those based on multi-sensor fusion and those based on single-vision.

Based on multi-sensor fusion, these methods aim to overcome the limitations of single sensors by combining information from different types of sensors to enhance detection robustness and accuracy. In the field of personnel safety detection, researchers have explored various fusion strategies. For example, to monitor the physiological and behavioral states of workers, Pham et al. [21] designed a real-time support system that fuses data from an inertial measurement unit (IMU), a barometer, and a carbon monoxide (CO) sensor. This approach can accurately identify a worker’s sudden fall or loss of physical performance, thereby effectively avoiding false alarms caused by insufficient information from a single sensor. Ma et al. [22] comprehensively assess the fatigue state of workers in elevated work environments by fusing data from multiple wearable sensors—such as heart rate, breathing, skin temperature, lactate and glucose from sweat, and electroencephalography (EEG)—to prevent fall-from-height accidents caused by fatigue. To address the issue of visual occlusion, Chen et al. [23] fused visual data with pressure signals from smart insoles to solve the challenge of 3D worker pose estimation in visually obstructed environments, combining it with external load data for a more comprehensive ergonomic assessment. Similarly, Wang and Yan [24] fused video images and surface electromyography (sEMG) signals to recognize workers’ operational behaviors; this method can assist in determining a worker’s behavioral state by analyzing their muscle activity signals when the camera is occluded and direct observation is not possible, thereby compensating for the monitoring blind spots of single-vision sensors. Furthermore, some researchers have extended the scope of fusion to the environmental level. For instance, Hyun Sim and Hyunwook Kim [25] proposed a multimodal data framework that combines video, audio, and environmental sensors (e.g., temperature, vibration), utilizing deep learning and reinforcement learning techniques to achieve a shift from traditional, static risk assessment to intelligent, real-time, predictive safety management.

However, the practical application of this method on offshore platforms still faces challenges. First, deploying and maintaining multiple sensors, especially wearable devices, is costly and complex to manage. Second, on platforms filled with metal pipes and steel structures, some sensor signals, such as wireless communication, are susceptible to multipath interference. Finally, the algorithms for synchronizing and fusing heterogeneous multimodal data are complex and demand high real-time computing resources. Therefore, considering cost-effectiveness and deployment feasibility, detection methods based on single-vision remain the most widely applied and mainstream research approach in this field.

Single-vision-based detection methods utilize cameras to capture image information, which is then analyzed by algorithms. Their main advantages lie in relatively low deployment costs, mature technology, and the ability to capture rich semantic information (such as personnel posture and clothing color). In recent years, computer vision tasks based on convolutional neural networks (CNNs), such as image classification and object detection, have become research hotspots, attracting widespread attention from both academia and industry [26]. Mainstream single-vision-based object detection algorithms can be broadly divided into single-stage and two-stage methods [27], with each approach having its own advantages in terms of detection speed and accuracy. In the task of worker safety monitoring on offshore oil platforms, it is crucial to ensure both high detection accuracy and rapid algorithmic response. Achieving efficient and reliable object detection in real-world environments holds significant research importance and practical value. The remote and hazardous nature of offshore drilling platforms, coupled with limited escape options, exacerbates the severity of accidents. This underscores the necessity of developing a reliable safety monitoring system that can accurately detect personnel and mitigate risks. The effectiveness of such a system largely depends on the performance of its underlying object detection algorithm.

Two-stage methods, such as Region-based Convolutional Neural Networks (R-CNNs) [28], Fast R-CNNs [29], and Faster R-CNNs [30], first generate a set of candidate regions before performing object classification and bounding box regression, which leads to higher accuracy. For example, Sun et al. [31] proposed an intelligent detection system for unsafe worker behavior based on deep learning, which uses a Faster R-CNN model to achieve real-time detection of such actions. This system improves detection accuracy, reduces the workload of safety supervisors, and effectively decreases the occurrence of safety accidents. However, because they must process each candidate region sequentially, these methods are generally slower, and their performance is limited in scenarios requiring high real-time performance.

As a widely used single-stage detection method, the YOLO series of algorithms has demonstrated significant potential in the field of industrial safety monitoring. By utilizing an anchor mechanism, it simultaneously performs object detection prediction and regression, thereby reducing the step of candidate region extraction and meeting the real-time detection requirements of various scenarios. Given this, most recent research on worker detection on offshore platforms has been based on the YOLO series of algorithms. Their evolution clearly reflects how researchers have progressively addressed the four core challenges of offshore platforms: “dynamic background noise,” “structural complexity,” “occlusion scenes,” and “environmental uncertainty”.

In early research, Gong et al. [18] proposed a personal protective equipment (PPE) detection method based on YOLOv3, combined with an improved random forest algorithm (RFA-YOLO), to enhance the localization and detection accuracy of workers and protective equipment. By constructing a dataset for offshore drilling platforms, experimental results showed that this method could quickly and accurately detect workers not wearing helmets or work clothes, making it particularly suitable for high-risk environments like offshore drilling platforms. Ji et al. [16] introduced a high-precision protective equipment detection framework based on an improved YOLOv4 version of the RFA-YOLO algorithm, specifically designed for offshore drilling platforms. This method combines object detection and classification techniques, using the improved YOLOv4 model to enhance the localization and detection capabilities for workers, helmets, and work clothes, while optimizing the detection process with a random forest algorithm. Salem et al. [32] proposed a real-time helmet detection system based on YOLOv8, designed for safety compliance checks in the oil and gas industry. The system accurately detects whether workers are wearing helmets, achieving an accuracy of 90.9%. This research showcases the potential of YOLOv8 in high-precision real-time monitoring, especially in safety monitoring tasks that require high accuracy and real-time performance. These studies achieved rapid detection of targets like helmets and work clothes by constructing specific datasets. However, they mainly focused on relatively clear scenarios, and their performance bottlenecks became apparent when dealing with severe occlusion and complex background interference, leading to prominent issues of missed detections and false positives.

To address the interference caused by the structural complexity of platform steel frames and pipes, as well as dynamic background noise from ocean waves, researchers began to focus on enhancing the model’s feature extraction and discrimination capabilities. Wang et al. [33] proposed a visual perception system for ship engine rooms based on YOLOv5. The system adopted an improved CSPDarknet53 network and a coordinate attention mechanism (C3CA) to enhance the detection of small objects (such as valves and instruments) in ship engine rooms. The modified YOLOv5 model improved the network structure, making it better suited for complex equipment layouts and multi-scale object detection. This study demonstrated the strong potential of YOLOv5 in dynamic scenes and for small object detection. However, its real-time processing capability in high-density, complex backgrounds still needs further improvement to address issues like multiple devices and occlusion in ship engine rooms. Yang et al., in their work [34], proposed the FE-YOLOv5 algorithm, which introduced a multi-scale feature enhancement module and bidirectional cross-scale connections (BiFPN), effectively improving the accuracy of small object detection. The algorithm performed exceptionally well in the low-light and complex background environments of drilling platforms, overcoming the limitations of traditional YOLO models in these conditions. Although FE-YOLOv5 performed well in specific scenarios, it still exhibited some detection latency when targets changed rapidly in extremely dynamic backgrounds, and its real-time response capability requires further optimization in the future.

Additionally, occlusion scenarios are a common challenge during intensive operations on offshore platforms. In the vast and structurally complex platform environment, workers often appear as small targets in surveillance footage and are easily occluded by equipment, pipes, or other colleagues. Li et al., in their paper [35], proposed BR-YOLO, an improved network based on YOLOv8, which enhances feature extraction for occluded targets by introducing a BiFormer module in the shallow layers of the network. To better understand worker intent under occlusion, Sun et al. [36] proposed a cascaded model that first utilizes YOLOv7-Pose for high-precision human pose estimation, and then uses an improved YOLOv7 model to detect the operational targets (e.g., valves, instruments) with which the worker is interacting. By establishing an association between pose and target, this method can not only determine if a worker is performing a valid operation but also infer their behavior based on their interaction with the target when parts of the body are occluded, providing a new approach to solving behavior analysis problems under occlusion.

Although these methods show potential in handling occlusion or specific targets, they are often optimizations for a single challenge and lack a unified framework that can systematically and balancedly address the four major challenges mentioned above. For example, high-precision helmet detection is not entirely equivalent to the robust detection of the entire human body under severe occlusion and dynamic backgrounds.

The YOLO series of algorithms is the benchmark for real-time object detection, and it has developed rapidly. For example, YOLOv7 [37] introduced innovations such as the Extended Efficient Layer Aggregation Network (E-ELAN), which significantly improved the model’s learning capability and detection efficiency without substantially increasing computational costs. Its successor, YOLOv8 [38], further surpassed existing levels by adopting a decoupled head, an anchor-free design, and a more flexible C2f module, achieving an excellent balance of speed and accuracy on general-purpose datasets.

However, despite their excellent performance as general-purpose detectors, these state-of-the-art models have inherent limitations when directly applied to the highly challenging environment of an offshore oil platform. First, their backbone networks, while powerful, are designed for general feature extraction. They lack specialized mechanisms to suppress the specific forms of noise prevalent on platforms, such as high-frequency structural noise from steel frames and pipes, and dynamic texture noise from ocean waves, which can easily obscure the features of worker targets. Second, the frequent and severe occlusion problems in the narrow workspaces of platforms pose a critical challenge to the default Non-Maximum Suppression (NMS) algorithm in these models. This traditional NMS is often too aggressive, permanently discarding bounding boxes with high overlap, which can lead to valid but partially occluded targets being incorrectly suppressed, resulting in a large number of missed detections.

It is precisely to address these core deficiencies of the latest general-purpose models within this specific industry context that we have undertaken this work. We clearly recognize the need for specialized adjustments to overcome these long-standing challenges, and this forms the basis for the improvements proposed in this paper.

YOLOv10 is a significant advancement in the YOLO series of real-time object detectors. It introduces key architectural improvements to optimize the balance between accuracy and response time. Through innovations such as a dual-head structure and more efficient feature fusion mechanisms, YOLOv10 enhances detection performance while maintaining low computational costs. This makes it a top-tier solution for real-time object detection tasks, establishing a new performance benchmark in the field.

Despite significant advancements in YOLO and other object detection algorithms across various domains, challenges remain in practical applications. In the task of small object detection in complex offshore environments, existing algorithms need to strike a balance between accuracy and real-time performance. Algorithms like YOLO and SSD still suffer from false positives and missed detections, especially in scenes with overlapping or high-density targets. The limitations of existing Non-Maximum Suppression (NMS) methods hinder their accuracy. Furthermore, transfer learning shows great potential in addressing data scarcity issues. By pre-training on large-scale datasets and transferring knowledge to the target task, the model’s generalization ability and adaptability can be improved. In application scenarios with scarce data, combining transfer learning with data augmentation techniques will further enhance the performance of object detection systems. To address these challenges, the PSD-YOLO algorithm proposed in this paper (based on YOLOv10) provides a detection framework for offshore oil platform worker detection that balances real-time performance and reliability, laying the foundation for building a more comprehensive and intelligent offshore platform monitoring system.

## 3. The Proposed PSD-YOLO Algorithm

**Figure 1:**
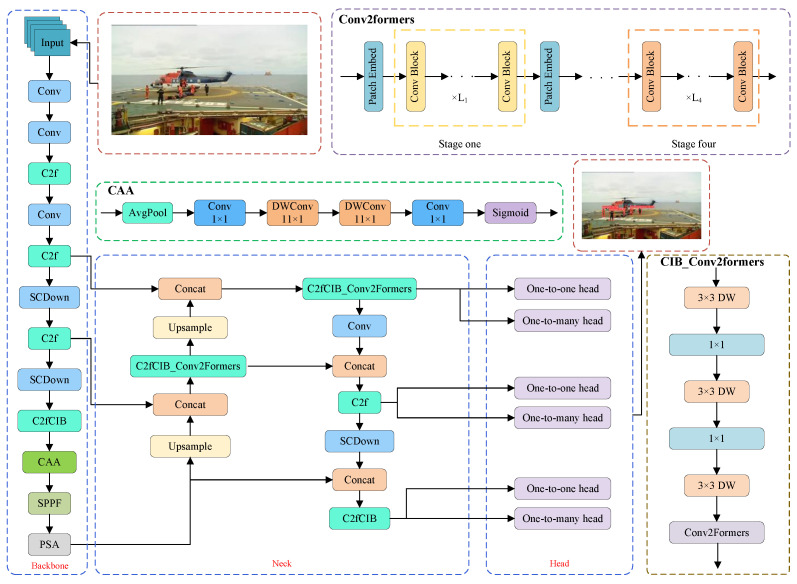
Architecture of the PSD-YOLO network for offshore oil platform worker detection.

PSD-YOLO is a novel object detection architecture specifically designed for the detection of workers on offshore oil platforms, balancing both real-time performance and accuracy. The architecture comprises a custom backbone, a redesigned neck for feature fusion, and an optimized detection head, as illustrated in Figure 1. The backbone network introduces a CAA module to enhance the model’s ability to capture long-range contextual information, which effectively suppresses complex background noise and improves focus on relevant targets. For the feature fusion neck, the newly designed C2fCIB_Conv2Former module replaces the two original C2f modules and utilizes large-kernel, depth-wise convolution to strengthen multi-scale feature fusion, optimize feature integration and modeling, and significantly boost performance, particularly for small-object detection. In the post-processing stage, the architecture employs the Soft-NMS algorithm to refine the box selection process, mitigating missed detections in scenarios with occluded targets and thereby improving both accuracy and robustness. Finally, the model’s practical effectiveness is enhanced through a strategy of Domain Adaptation via Cross-Domain Knowledge Transfer. This approach begins by initializing the model with weights from a YOLOv10s model pre-trained on the large-scale COCO dataset, leveraging robust, generalized feature representations as a strong prior. A staged fine-tuning process then freezes the base network layers to retain these generic capabilities while training deeper layers to specialize in the target domain’s unique features, such as specific work attire and complex industrial backgrounds. This not only shortens training time but also significantly improves detection accuracy and efficiency under real-world platform conditions, effectively mitigating the risk of overfitting associated with a limited specialized dataset. The effectiveness of each of these structural and strategic optimizations, and the quantifiable ‘gain’ they provide, is individually validated in our ablation study.

### 3.1. The CAA Module

The operating environment of offshore oil platforms is complex, with workers often blending into the surrounding background facilities, leading to significant background noise in images. This background noise is not merely random but includes significant structured interference from intersecting steel frames and pipelines, as well as dynamic textural noise from sea waves, both of which are easily confused with worker silhouettes. To address this specific challenge in a targeted manner, this paper incorporates the CAA module. Its architecture is particularly effective at capturing long-range contextual information, which is crucial for disambiguating worker silhouettes from the visually similar linear structures prevalent in the background. By dynamically adjusting the importance of target features, the CAA module allows the model to more precisely focus on the target and improve detection accuracy in the presence of this complex, domain-specific noise [39].

**Figure 2:**
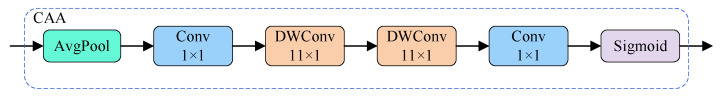
The architecture of the CAA module.

The core idea of the CAA module is to emphasize local features through weighted input features while enhancing the representation of local regions by integrating contextual information. Figure 2 illustrates the structure of the CAA module. First, an average pooling operation is applied to the input feature matrix, followed by further processing of local features using a 1 × 1 convolution to refine local details, which is formulated as follows:

(1) $$F_{avg}=AvgPool\left(X^{\left(in\right)}\right)$$

(2) $$F_{local}=Conv1\times 1\left(F_{avg}\right)$$

where $X^{\left(in\right)}$ denotes the input feature map, $F_{avg}$ represents the pooled feature map, and AvgPool refers to the average pooling operation. This step serves to refine the features, thereby providing more effective local information for the subsequent depth-wise convolution operations.

In the feature extraction stage, the CAA module applies Depthwise Separable Convolution [40] to the input features in both vertical and horizontal directions to extract multi-directional fine-grained features, thereby effectively capturing the morphological features of the target. The following formula is used:

(3) $$F_{width}=DepthwiseConv_{vert}\left(F_{local}\right)$$

(4) $$F_{height}=DepthwiseConv_{hor}\left(F_{width}\right)$$

where $DepthwiseConv_{vert}$ and $DepthwiseConv_{hor}$ denote the Depthwise Separable Convolution applied in the vertical and horizontal directions, respectively, and $F_{width}$ and $F_{height}$ are the features obtained through convolution in the width and height directions, respectively. This process not only enhances the feature representation of the target but also mitigates background noise interference, leading to improved detection accuracy.

The CAA module introduces a dynamic attention mechanism after feature generation, which adjusts the feature intensity in the target and background regions by producing an attention map. The generated attention map A is non-linearly transformed by a sigmoid activation function to adjust the weight distribution between the target and background, thereby effectively suppressing background noise, as shown in the following formula:

(5) $$A=\sigma (Conv1\times 1\left(F_{height}\right))$$

where *A* represents the generated attention map and σ denotes the sigmoid function. Finally, the enhanced features are fused with the input features through residual connections, which further enhance the model’s robustness in complex scenarios, as expressed in the following equation:

(6) $$F_{enhanced}=A⊙X^{\left(in\right)}$$

The CAA module performs context-aware enhancement of deep features through an adaptive feature weight assignment mechanism. By integrating the CAA module with the C2fCIB module in the backbone network, a hierarchical, higher-order feature enhancement architecture is established to improve the model’s deep semantic features, thereby enhancing background noise suppression and target area focusing.

### 3.2. C2fCIB_Conv2Former Module

**Figure 3:**
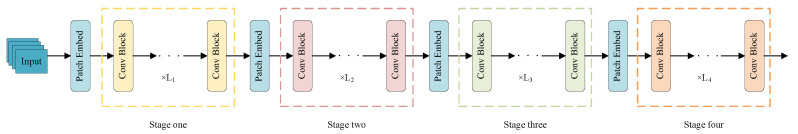
Structure of the Conv2Former architecture with patch embedding and convolution blocks.

Due to interference from waves, weather conditions, and the platform’s background, worker targets often exhibit low pixel occupancy and unclear texture information. To enhance the model’s detection performance for weak targets, this paper introduces the C2fCIB_Conv2Former module. This architectural optimization improves the fusion capability of multi-scale features by incorporating large kernel depthwise convolutions, thus, improving the model’s ability to integrate features in complex environments. As illustrated in Figure 3, the Conv2Former [41] module consists of four stages, with the Patch Embedding module inserted between each pair of successive stages to reduce spatial resolution, leading to reduced computational overhead when processing high-resolution images, compared to traditional convolutions.

**Figure 4:**
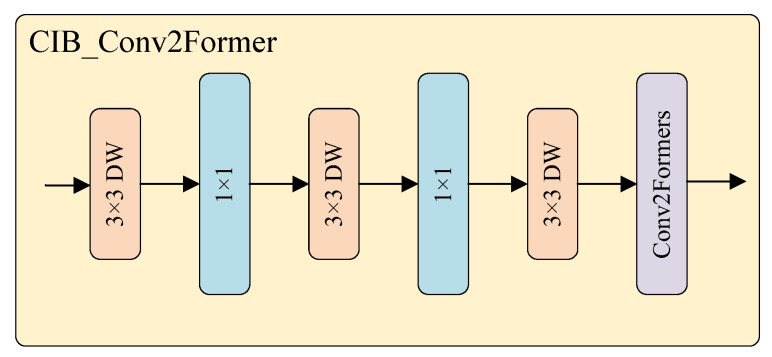
Architecture of the proposed CIB_Conv2Former block, the core component within the C2fCIB_Conv2Former module.

As shown in Figure 4, the C2fCIB_Conv2Former module is designed by incorporating the Conv2Former module into the C2fCIB structure. Leveraging Depthwise Separable Convolution and Convolution Modulation mechanisms [42], the Conv2Former module accurately models long-range dependencies to enhance the model’s global context modeling capability. This optimization is particularly effective for detecting small and occluded targets in complex environments, improving overall detection robustness by reducing false detections and omissions.

**Figure 5:**
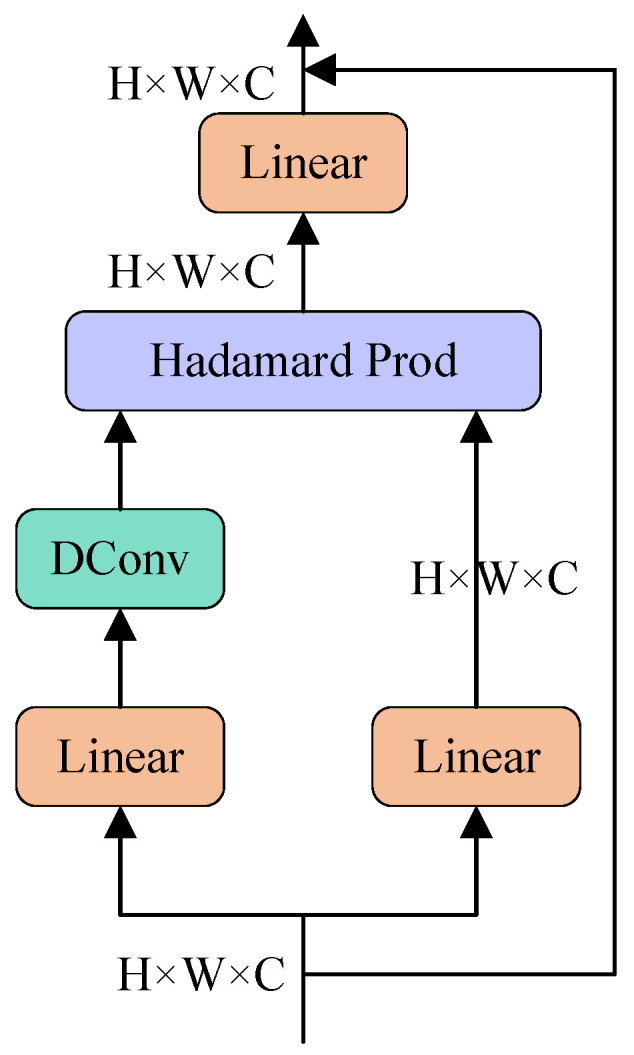
Architecture of the convolutional modulation operation in the Conv2Former module.

The core method of the Conv2Former module is the convolutional modulation operation depicted in Figure 5. The input features first pass through two independent linear layers, where one output is processed by depthwise convolution (DConv) [43], and the other output undergoes the Hadamard product operation. The result is then passed through another linear layer and finally modulated with the original input features to generate the output. Compared to Transformer, which increases computational complexity by the square of the resolution, this operation increases complexity linearly, thus, effectively reducing the computational cost.

In this process, the convolutional modulation operation dynamically adjusts feature weights via the Hadamard product. This enables the model to focus on salient regions while mitigating interference from background noise, ultimately improving detection performance. Given an input $X\in R^{H\times W\times C}$, the convolutional modulation operation can be expressed as follows:

(7) $$\begin{array}{cc} A & =DConv_{k\times k}\left(W_{1}X\right) \end{array}$$

(8) $$\begin{array}{cc} V & =W_{2}X \end{array}$$

(9) $$\begin{array}{cc} Z & =A⊙V \end{array}$$

where ⊙ denotes the Hadamard product, $W_{1}$ and $W_{2}$ represent the weight matrices of the two linear layers, $DConv_{vtimesk}$ refers to the depthwise convolution and corresponding operation, while *A* and *V* represent the feature maps after the convolution and linear transformation, respectively. The final output, the augmented feature map, is obtained through this operation, yielding *Z*.

The Conv2Former module emphasizes the interaction and correlation of global features. By combining with the C2fCIB module, the C2fCIB_Conv2Former module is integrated into the neck network and reused multiple times, effectively enhancing the fusion capability of multi-scale features, improving cross-scale target detection accuracy, and boosting the model’s ability to detect small targets in complex scenes.

### 3.3. Soft-NMS Algorithm

To address this problem, this paper employs an algorithmic optimization by adopting the Soft-NMS [44] algorithm in place of traditional NMS, which mitigates the target leakage problem caused by overlapping frames.

The traditional NMS algorithm suppresses redundant bounding boxes by directly setting the score of overlapping boxes to zero. The calculation is expressed as follows:

(10) $$S_{i}=\left\{\begin{array}{c} S_{i},\mathrm{iou}\left(M,b_{i}\right)<N_{t}\\ 0,\mathrm{iou}\left(M,b_{i}\right)\geq N_{t} \end{array}\right.$$

where $\mathrm{iou}(M,b_{i})$ represents the degree of overlap between frame *M* and the detection frame $b_{i}$, $N_{t}$ is the predetermined overlap threshold, and $S_{i}$ is the score of the current detection frame.

A key limitation of standard NMS is its tendency to erroneously discard valid detection boxes in scenarios with high object overlap. To address this, we employ the Soft-NMS algorithm. Unlike traditional NMS, which sets the score of an overlapping box to zero, Soft-NMS applies a penalty function to decay the detection score of a box $b_{i}$ based on its Intersection over Union (IoU) with the highest-scoring detection box *M*. This method avoids aggressive suppression and improves recall in dense scenes.

The core idea of Soft-NMS is that, when dealing with overlapping frames, it does not completely set their scores to zero, but gradually decays the scores according to the degree of overlap with the current frame. For example, if a frame overlaps with the highest scoring frame *M*, its score is not directly set to zero but is adjusted according to its overlap with *M* using a decay function. If the overlap is small, the score undergoes less decay; if the overlap is large, the score undergoes more decay. This process is represented by the following formula:

(11) $$S_{i}=\left\{\begin{array}{cc} S_{i}, & \mathrm{if}\;\mathrm{iou}\left(M,b_{i}\right)<N_{t}\\ S_{i}\left(1-\mathrm{iou}\left(M,b_{i}\right)\right), & \mathrm{if}\;\mathrm{iou}\left(M,b_{i}\right)\geq N_{t} \end{array}\right.$$

Using this method, Soft-NMS effectively reduces false suppression in cases of multiple overlapping frames, improving overall detection accuracy and stability. In particular, when partial occlusion of the target occurs, Soft-NMS can retain potential target frames to avoid missed detections, thereby improving the model’s robustness in complex environments.

In addition, the computational complexity of the Soft-NMS algorithm is comparable to that of the traditional NMS algorithm, $O(N^{2})$, and does not introduce additional computational burden in practical applications. By attenuating detection frame scores instead of removing them completely, the algorithm maintains high detection efficiency while ensuring accuracy, making it suitable for the dynamic and complex environment of offshore oil platforms. In our implementation, the overlap threshold $N_{t}$ is set to 0.5 to achieve an optimal balance between suppressing redundant boxes and retaining detections for occluded objects.

### 3.4. Domain Adaptation via Cross-Domain Knowledge Transfer

Transfer learning [45] is a training paradigm that transfers knowledge learned from a source domain for use in a target domain. To address the dual challenges of data scarcity and the unique environmental complexities of offshore platforms, we employ a tailored knowledge transfer strategy for robust model adaptation. Our approach begins by initializing the model with weights from YOLOv10s pre-trained on the large-scale COCO dataset. This strategy serves not merely to accelerate convergence, but more critically, to transfer foundational knowledge, allowing the model to leverage robust, generalized feature representations of human silhouettes and structures learned from a vast and diverse source domain as a strong prior.

A staged fine-tuning process is then implemented by freezing the base network layers to retain the generic representation capabilities acquired from the source domain. Meanwhile, the deeper layers are trained to enable the model to specialize in the target domain’s unique features, such as specific work attire and complex industrial backgrounds, thereby enhancing feature discriminability while preserving prior knowledge.

## 4. Experiments Analysis

### 4.1. Data Set

To ensure a high degree of alignment between model training and practical application, we constructed a dataset entirely from real-world operational scenarios on offshore oil platforms. Compared to existing general-purpose pedestrian detection datasets, such as COCO and CityPersons, our dataset offers irreplaceable advantages. General-purpose datasets primarily feature common scenarios like urban streets and indoor environments, whereas our dataset focuses on the unique and complex industrial setting of offshore platforms. Its sample uniqueness is characterized by specific challenges such as dynamic sea wave backgrounds, dense occlusions from steel structures, distinctive work attire, and variable marine lighting conditions—all of which are absent or underrepresented in general-purpose datasets.

The dataset was built from operational videos of offshore platforms collected from multiple sources. As the footage originates from various operational teams and scenarios, specific model details of the UAVs and handheld devices used for filming were not available with the source videos. The final dataset consists of 13,202 high-quality images with a uniform resolution of 960 × 540, non-uniformly covering four typical challenging scenarios: small targets against complex backgrounds, low-light environments, background interference from sea waves, and severe target occlusion in narrow spaces. After a meticulous manual curation process, the images were annotated with bounding boxes according to a strict protocol.

To ensure the reliability of annotation quality, we established detailed annotation rules. These rules required that bounding boxes tightly enclose the entire visible body outline of a worker, rather than only annotating specific parts such as the head or torso, to ensure localization precision. To ensure consistency and minimize issues such as annotation offset, we implemented a two-stage cross-validation mechanism for quality control. Specifically, each image was first preliminarily annotated by one annotator, after which it was reviewed and corrected by other team members. Any controversial annotations were resolved through team discussion to ensure the high consistency and accuracy of the final results.

The dataset was randomly split into a training set (9902 images) and a validation set (3300 images) following a strict 3:1 ratio. We applied data augmentation techniques such as image flipping and brightness attenuation to the training set to improve the model’s generalization capability and robustness. The self-built dataset used in this study is available from the corresponding author upon reasonable request.

**Figure 6:**
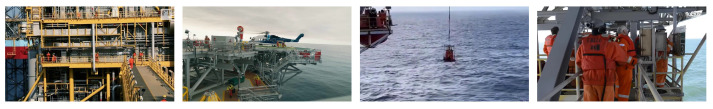
Representative samples from the offshore oil platform worker detection dataset.

Representative samples of four typical scenarios are shown in Figure 6. The first figure shows small targets under complex backgrounds, which often result in inaccurate localization. The second figure shows large-scale targets in low-light environments, which are prone to missed detections. The third figure shows targets affected by wave interference, leading to both missed detections and localization errors. The fourth figure shows occluded targets in narrow working spaces, where occlusion easily causes missed detections.

### 4.2. Environment Setup

All experiments were conducted on a server running the Ubuntu operating system with the PyTorch deep learning framework. The detailed hardware and software configurations are shown in Table 1.

To ensure fairness and reproducibility, a consistent set of hyperparameters was used for training all models. The key hyperparameter settings, such as the optimizer, learning rate strategy, and batch size, are listed in Table 2.

### 4.3. Evaluation Metrics

In this paper, a set of evaluation metrics, including Giga Floating Point Operations (GFLOPs), mean Average Precision (mAP), Precision (P), Recall (R), and Frames Per Second (FPS), is used to assess the model’s performance.

GFLOPs is a metric used to measure the computational complexity of the model. mAP is a widely used and comprehensive metric for evaluating object detection models. It is computed by first calculating the Average Precision (AP) for each class and then averaging the AP values across all classes. P measures the proportion of correctly predicted positive samples among all predicted positives, reflecting the model’s accuracy in positive predictions. R measures the proportion of correctly predicted positive samples among all actual positives, reflecting the model’s sensitivity or completeness in identifying true objects. The evaluation formulas are defined as follows:

(12) $$\begin{array}{cc} P & =\frac{\mathrm{TP}}{\mathrm{TP}+\mathrm{FP}} \end{array}$$

(13) $$\begin{array}{cc} R & =\frac{\mathrm{TP}}{\mathrm{TP}+\mathrm{FN}} \end{array}$$

(14) $$\begin{array}{cc} \mathrm{AP} & =\int_{0}^{1}P\;dR \end{array}$$

(15) $$\begin{array}{cc} \mathrm{mAP} & =\frac{1}{N}\sum_{i=1}^{N}{\mathrm{AP}}_{i} \end{array}$$

where TP is the number of true positives, FP is the number of false positives, FN is the number of false negatives, ${\mathrm{AP}}_{i}$ is the average precision for the i category, and N is the total number of categories. FPS refers to the processing speed of the model, indicating the number of frames that can be processed per unit of time. The formula for calculating FPS is as follows:

(16) $$\mathrm{FPS}=\frac{1000}{T_{\mathrm{pre}}+T_{\inf}+T_{\mathrm{post}}}$$

$T_{pre}$ is the pre-processing time, $T_{inf}$ is the inference time, $T_{post}$ is the post-processing time.

### 4.4. Analysis of Experimental Results

#### 4.4.1. Ablation Study

To validate the rationality of the proposed model, this study conducts ablation experiments on the transfer learning, Soft-NMS, CAA, and C2fCIB_Conv2Former modules in the PSD-YOLO algorithm under identical parameter settings. We use an unmodified YOLOv10s as our baseline model, with its performance presented in the first row (Model 1) of Table 3, and incrementally add our proposed enhancements. The results of the ablation experiments are presented in Table 3, where the best-performing result for each metric is highlighted in bold and the second-best is marked with an underline. After introducing the transfer learning strategy into the YOLOv10s model, the model’s precision and recall improved by 1.2% and 0.2%, respectively. This is because transfer learning utilizes pre-trained human contour features from large-scale datasets, enhancing the model’s detection accuracy for workers in complex environments. To address missed detections due to personnel occlusion in constrained workspaces, the Soft-NMS module is designed to progressively reduce the scores of overlapping boxes, increasing the model’s average precision by 0.5% without additional computational cost. The CAA module, introduced after the C2fCIB in the backbone network, enhances the model’s ability to capture long-range contextual information. Finally, the C2fCIB_Conv2Former module, designed with large-kernel depth-wise convolution to enhance multi-scale feature fusion, increases the average precision by 0.6% while reducing the model’s computational complexity by 4.0%. With acceptable inference time overhead, the PSD-YOLO algorithm proposed in this study effectively improves the model’s detection accuracy.

#### 4.4.2. Comparative Experiments

**Figure 7:**
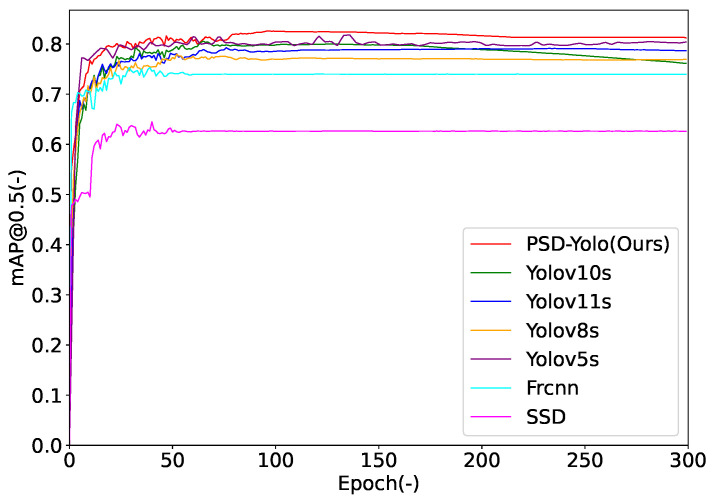
mAP@0.5 curves of different models during training on the offshore oil drilling platform worker detection dataset.

(1) Performance Validation of the C2fCIB_Conv2Former Module
  To validate the effectiveness of our proposed C2fCIB_Conv2Former module, we conducted a comparative analysis. We use the original YOLOv10s-based model containing only the basic C2fCIB structure as our baseline, with its performance presented in the first row of Table 4. Our module and several other state-of-the-art attention mechanisms were integrated into the same baseline network architecture for a fair comparison. The competing modules include Monte Carlo Attention (MCAttn) [46], Dynamic Convolution (DynamicConv) [47], Single-Head Self-Attention (SHSA) [48], BiFormer (BiF) [49], and Patch-Aware Attention (PPA) [50]. The detailed comparison results are presented in Table 4. The best result is highlighted in bold.
  As shown in Table 4, the C2fCIB_Conv2Former model demonstrates superior detection accuracy in worker detection tasks under complex offshore oil drilling platform backgrounds, while also reducing the computational load of the baseline model. Compared to other mainstream C2fCIB modules, the model based on the C2fCIB_Conv2Former module achieves a higher mAP@0.5 of 81.3%, a recall of 73.2%, and a precision of 89.1%, surpassing other models. Through the aforementioned architectural improvement, the C2fCIB_Conv2Former module effectively improves the model’s detection accuracy for small targets in complex backgrounds and occlusion scenarios, demonstrating its potential for practical deployment.
(2) Performance Validation of the CAA Module
  To validate the effectiveness of the CAA module in improving the detection performance of workers on offshore oil platforms, we compare it with several mainstream attention mechanisms, including Squeeze-and-Excitation (SE) [51], a Convolutional Block Attention Module (CBAM) [52], Efficient Channel Attention (ECA) [53], Coordinate Attention (CA) [54], A Simple Parameter-Free Attention (SimAM) [55], a Global Attention Mechanism (GAM) [56], Efficient Multi-Scale Attention (EMA) [57], ShuffleAttention [58], and a Normalization-based Attention Module (NAM) [59]. We use the unmodified YOLOv10s model as our baseline, with its performance presented in the first row of Table 5, to evaluate the performance changes after integrating different attention modules. The detailed comparison results are presented in Table 5.
  As shown in Table 5, the CAA module demonstrates significant advantages in the worker detection task on offshore oil platforms. Compared to mainstream attention mechanisms, the model’s detection accuracy achieves the highest value among all compared methods, indicating that CAA can capture targets more comprehensively and enhance multi-scale detection robustness. Specifically, the CAA model achieves an mAP@0.5 of 0.817, showing a significant improvement over other attention mechanisms. Furthermore, the CAA model exhibits an excellent recall of 0.736, indicating its superior ability to detect targets in complex background environments.
  Although its inference speed is slightly lower than the baseline YOLOv10s model, it outperforms most attention-based methods. Compared to other attention mechanisms, it is noteworthy that while ShuffleAttention and GAM are close to CAA in mAP@0.5, their mAP@0.5-0.95 scores are 1.1% and 0.9% lower, respectively, highlighting CAA’s stronger adaptability to complex scenarios. Overall, by optimizing the channel attention mechanism, CAA significantly enhances detection performance while ensuring real-time capability, making it particularly suitable for the precise identification of small targets in dynamic offshore environments.
(3) Comparative Experiments with Mainstream Algorithms
  To objectively evaluate the detection performance of the model, this study compares PSD-YOLO with several mainstream detection models on the offshore oil drilling platform worker detection dataset, including the two-stage object detection algorithm Faster R-CNN with high detection accuracy and single-stage object detection algorithms SSD, YOLOv5s, YOLOv8s, YOLOv10s, and YOLOv11s.
  The detection results are presented in Table 6. Although single-metric winners, their performance in other key areas is significantly lower, making them unsuitable for this balanced task. In contrast, our proposed PSD-YOLO not only achieves the highest detection accuracy (mAP@0.5) and recall, but also secures competitive, second-best results in precision. This demonstrates that PSD-YOLO strikes a superior balance between accuracy and efficiency, making it a highly effective and robust solution for personnel detection in dynamic marine environments. As seen in the FPS metrics in Table 6, while models like YOLOv5s have a higher raw speed, their accuracy is lower; PSD-YOLO, while achieving a significant lead in mAP, maintains an inference speed of 232.56 FPS, which fully meets the requirements for real-time monitoring.
  Figure 7 illustrates the mAP@0.5 convergence curves of the different models during the training process. From the trend of the curves, it can be observed that PSD-YOLO converges the fastest and exhibits the most stable performance. Within the first 50 epochs, its mAP value rises rapidly and stabilizes around 100 epochs, outperforming all other models. In comparison, while the mAP values of YOLOv5s, YOLOv8s, YOLOv10s, and YOLOv11s are also relatively high, they remain slightly lower than PSD-YOLO in the final stages of training, indicating a weaker adaptability to this complex detection task. The mAP curves of SSD and Faster R-CNN remain at a low level throughout the training process. Specifically, the mAP of SSD rises rapidly in the early stages of training and then stabilizes around 0.6, while the mAP of Faster R-CNN stabilizes around 0.74, indicating the limited capability of these two models in handling complex object detection tasks.

#### 4.4.3. Visualization Results

**Figure 8:**
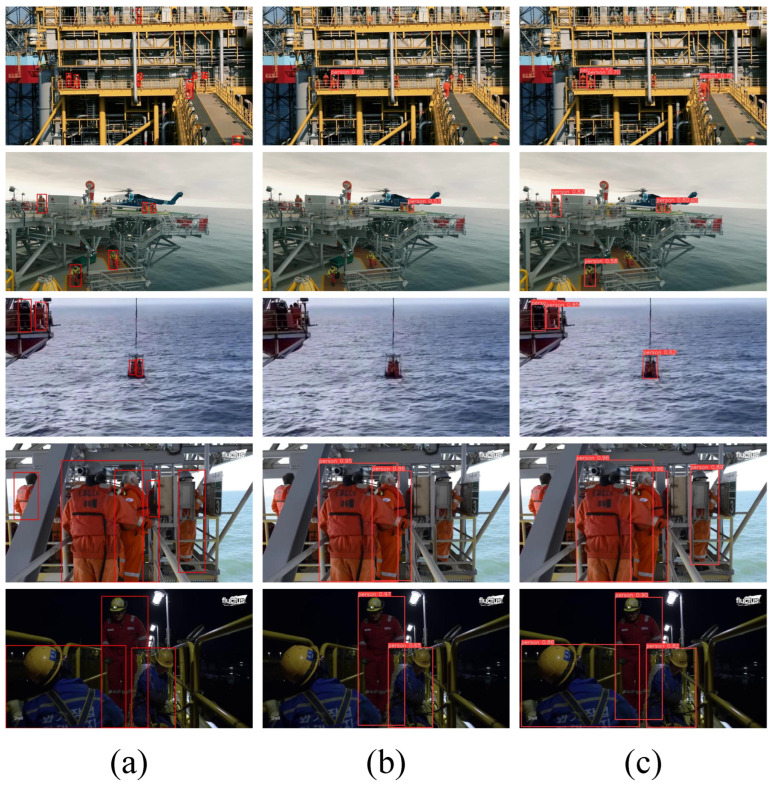
Detection results of YOLOv10s and PSD-YOLO in five typical offshore oil platform scenarios: (**a**) Ground Truth, (**b**) YOLOv10s, (**c**) PSD-YOLO.

To visually demonstrate the superiority of the proposed PSD-YOLO algorithm, Figure 8 presents a qualitative comparison of its detection results against the baseline YOLOv10s across five typical challenging scenarios on offshore platforms. In the figure, column (a) is the Ground Truth, column (b) shows the results from YOLOv10s, and column (c) displays the results from PSD-YOLO. Specifically, in the first row featuring a complex background of platform steel structures, the baseline model (b) misses several distant workers on the upper walkway, whereas PSD-YOLO (c) identifies relatively more of the distant workers. For the small targets in the second row, such as the distant helicopter and the person beside it, the baseline model (b) fails to detect them entirely, while PSD-YOLO (c) accurately localizes both, showcasing its superior small-object detection capability. Similarly, amidst interference from the sea wave background in the third row, the baseline model (b) fails to recognize the worker involved in the lifting operation and another worker on the adjacent platform, while PSD-YOLO (c) successfully detects them, indicating better robustness to dynamic background noise. When faced with a group of workers with severe mutual occlusion in the fourth row, the baseline model (b) only identifies some of the individuals; in contrast, PSD-YOLO (c), leveraging the improved Soft-NMS algorithm, more accurately detects a greater number of partially occluded workers. Finally, in the fifth row’s nighttime scenario with low light and low contrast, the baseline model (b) misses a detection, whereas PSD-YOLO (c) identifies more of the workers. In summary, these visual comparisons clearly demonstrate the significant advantages of PSD-YOLO in handling key challenges.

## 5. Discussion

### 5.1. Interpretation of Results and Comparison with Related Work

The proposed PSD-YOLO model demonstrated exceptional performance on our custom dataset, achieving an mAP@0.5 of 82.5% and surpassing the YOLOv10s baseline and other mainstream algorithms. This result is significant because it provides effective solutions to several key challenges identified in previous research. For instance, the YOLOv4-based framework by Ji et al. [16] had limited performance when facing complex backgrounds and target occlusion, while the ACD-Net by Li et al. [20] also required improvement under adverse conditions and for occluded targets. Our model directly responds to these issues with two key designs: first, the integrated CAA module significantly enhances the model’s ability to distinguish workers from the platform’s steel structure background through its efficient long-range contextual modeling, effectively resolving the problem of background interference. Second, the application of the Soft-NMS algorithm, as confirmed by the ablation study, effectively improves the detection recall rate in crowded or occluded scenes, compensating for the shortcomings of the aforementioned studies.

Furthermore, while the work by Gong et al. [18] based on YOLOv3 achieved high accuracy, it was noted to have room for improvement in multi-scale feature fusion for occluded targets. Our newly designed C2fCIB_Conv2Former module was created precisely to address this issue; it utilizes large-kernel depth-wise convolution to optimize feature fusion for both small, distant targets and larger, closer ones, ensuring robust detection across multiple scales. Therefore, PSD-YOLO not only surpasses previous research in overall performance but, more importantly, provides a clear and verifiable paradigm. In contrast to state-of-the-art general-purpose detectors like YOLOv8, which provide a strong baseline but are not inherently optimized for the specific challenges of offshore platforms, our results demonstrate that the targeted enhancements in PSD-YOLO—such as the CAA module for noise suppression and Soft-NMS for occlusion—directly address the key failure points of these standard models in the platform’s high-clutter and high-density environments. This marks a clear advancement for this specific application by transforming a powerful general-purpose detection framework into a specialized model adapted for a specific, high-risk industrial scene through a series of modular, targeted improvements.

### 5.2. Limitations and Practical Deployment Challenges

**Figure 9:**
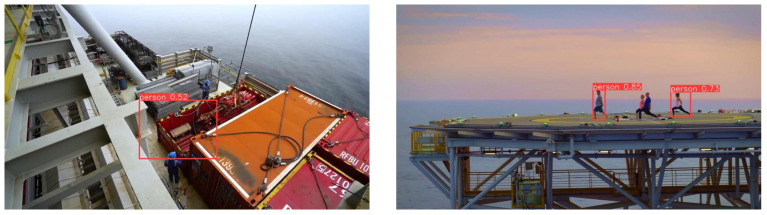
Example results of false detection.

Despite the encouraging results, several limitations of PSD-YOLO must be acknowledged. First, as noted in the dataset description, the dataset used in this study has an imbalanced sample distribution. Specifically, instances of “target occlusion” and “low-light environments” are significantly underrepresented compared to the more prevalent scenarios of “dynamic background” and “complex structure background.” This imbalance is an acknowledged limitation, as it may constrain the model’s generalization performance and robustness in these underrepresented conditions. Future work should, therefore, focus on augmenting the dataset for these specific scenarios—either through targeted data collection or advanced data augmentation techniques—to create a more balanced training set and further enhance the model’s overall performance. Second, the dataset was primarily collected under relatively clear weather and does not sufficiently cover extreme adverse conditions such as dense fog or heavy rain, nor does it include samples from challenging lighting like intense glare at sunrise/sunset or near-darkness with partial artificial lighting. Consequently, the model’s robustness under these unseen, extreme conditions remains to be verified. Furthermore, the model is still prone to certain recognition errors, and its ability to generalize to unseen appearances is limited, as illustrated in Figure 9. For instance, even when targets are in standard work attire, complex industrial background interference can lead to a combination of recognition errors, including missed detections, severely inaccurate localization, and very low confidence scores. Concurrently, when a target is wearing casual attire that differs significantly from the work clothes common in the training data, the model may miss the detection due to feature mismatch. These cases indicate that further enhancing the model’s robustness and generalization capabilities in open-world scenarios is a key focus for our future work. Furthermore, the performance improvements reported in this study are based on standard metrics like mAP, but formal statistical significance tests were not conducted, which is an area for refinement in future research.

Furthermore, deploying PSD-YOLO in a real-world offshore environment presents formidable engineering challenges. Our model achieves high frame rates and accuracy on an NVIDIA RTX 3090, but its real-time performance on resource-constrained edge computing devices commonly used on offshore platforms, such as the NVIDIA Jetson series, requires further optimization and validation through techniques like model quantization. Additionally, the camera hardware deployed on the platform must be ruggedized to withstand the harsh marine environment, including salt spray corrosion, high humidity, and constant vibrations. The long-term stable operation of the model also necessitates an effective maintenance and update mechanism to counteract potential “concept drift” caused by changes in platform layout or personnel attire.

### 5.3. Future Work

To address these limitations and further enhance the system’s intelligence, our future work will focus on several key directions. First, we will aim to improve the model’s adaptability to all-weather and all-lighting conditions. A highly promising avenue is multi-modal data fusion, for instance, combining data from infrared or thermal cameras with traditional RGB visual data to leverage the complementary strengths of different sensors, thereby overcoming the challenges posed by low-visibility environments such as dense fog and darkness. Second, to address the issues of generalization and “concept drift,” we will investigate continual learning and domain adaptation techniques. This would enable the model to continuously learn from new data post-deployment, maintaining its long-term effectiveness as the on-site environment evolves. Finally, our long-term objective is to extend this research from simple object detection to more complex behavior analysis. We plan to develop algorithms capable of real-time recognition of abnormal events such as worker falls, entry into restricted zones, or unsafe operations, thus, elevating the monitoring system from passive “detection” to proactive “warning” and providing a deeper level of intelligent assurance for safety on offshore platforms.

## 6. Conclusions

To address the challenges of poor small-object detection and occlusion in complex offshore platform environments, this paper proposes the PSD-YOLO algorithm for recognizing offshore workers. By incorporating the CAA module and the C2fCIB_Conv2Former module, the model significantly enhances its ability to capture long-range contextual information and fuse multi-scale features, leading to a substantial improvement in detection performance, particularly in scenarios involving small objects and significant background noise. The enhanced Soft-NMS algorithm effectively mitigates the missed detection problem caused by object occlusion, while the use of transfer learning not only accelerates the model’s training convergence but also boosts its adaptability and robustness in complex offshore environments. Comprehensive comparative experiments and ablation studies validate the effectiveness of the proposed algorithm, demonstrating its strong application potential for offshore drilling platforms. In future research, we will further focus on enhancing the model’s detection accuracy and stability under harsh weather conditions.

## Figures and Tables

**Table 1:** Experimental Setup.

Component	Specification
Operating System	Ubuntu 20.04
GPU	NVIDIA GeForce RTX 3090 (24 GB)
CPU	15 vCPU Intel® Xeon® Platinum 8362 @ 2.80 GHz
Framework	PyTorch 1.11, CUDA 11.3

**Table 2:** Training hyperparameter settings.

Hyperparameter	Value
Optimizer	Stochastic Gradient Descent (SGD)
Input Image Size	960 × 540
Batch Size	8
Total Epochs	300
Initial Learning Rate	0.01
Learning Rate Scheduler	Cosine annealing
Learning Rate Decay Factor	0.937
Momentum Factor	0.937
Weight Decay	0.0005
Warm-up Phase	20 epochs

**Table 3:** Ablation study results of the PSD-YOLO algorithm. TL: Transfer learning, S-NMS: Soft-NMS, C2C-CF: C2fCIB_Conv2Former. Our proposed model and the best result are highlighted in bold, and the second-best result is underlined.

Model	TL	S-NMS	CAA	C2C-CF	mAP@0.5	P	R	FPS	GFLOPs
1	—	—	—	—	0.805	0.875	0.721	**285.71**	24.4
2	🗸	—	—	—	0.806	0.887	0.723	270.27	24.8
3	🗸	🗸	—	—	0.81	0.881	0.731	270.27	24.8
4	🗸	🗸	🗸	—	0.819	0.892	0.732	270.27	25.2
5	🗸	🗸	🗸	🗸	**0.825**	**0.895**	**0.744**	232.56	**24.2**

**Table 4:** Comparative results of different attention mechanisms combined with C2fCIB on the offshore platform worker detection dataset. Our proposed model and the best result are highlighted in bold, and the second-best result is underlined.

Model	P	R	mAP@0.5	mAP@0.5-0.95	FPS	GFLOPs
C2fCIB (Baseline)	0.875	0.721	0.805	0.538	285.71	24.4
C2fCIB_MCAttn [46]	0.889	0.709	0.794	0.538	256.41	24.9
C2fCIB_DynamicConv [47]	**0.892**	0.728	0.810	0.556	**294.12**	24.4
C2fCIB_SHSA [48]	0.890	0.722	0.805	0.548	**294.12**	**23.8**
C2fCIB_BiF [49]	0.882	0.729	0.812	0.555	285.71	**23.8**
C2fCIB_PPA [50]	0.884	0.727	0.801	0.550	250.00	26.3
**C2fCIB_Conv2Former**	0.891	**0.732**	**0.813**	**0.558**	270.27	24.0

**Table 5:** Comparative results of different attention mechanisms for worker detection on the dataset. Our proposed model and the best result are highlighted in bold, and the second-best result is underlined.

Model	P	R	mAP@0.5	mAP@0.5-0.95	FPS
YOLOv10s (Baseline)	0.875	0.721	0.805	0.538	**285.71**
SE [51]	0.876	0.727	0.809	0.539	256.41
CBAM [52]	0.873	0.703	0.787	0.524	270.27
ECA [53]	0.872	0.721	0.805	0.533	**285.71**
CA [54]	0.883	0.713	0.804	0.531	**285.71**
SimAM [55]	0.883	0.711	0.799	0.529	256.41
GAM [56]	0.887	0.724	0.813	0.541	256.41
EMA [57]	0.881	0.715	0.797	0.53	270.27
ShuffleAttention [58]	0.877	0.722	0.813	0.539	**285.71**
NAM [59]	0.881	0.703	0.785	0.528	270.27
**CAA**	**0.888**	**0.736**	**0.817**	**0.55**	256.41

**Table 6:** Comparative results of PSD-YOLO with mainstream detection algorithms on the offshore oil drilling platform worker detection dataset. Our proposed model and the best result are highlighted in bold, and the second-best result is underlined.

Model	P	R	mAP@0.5	FPS	GFLOPs
SSD	**0.909**	0.470	0.670	38.35	30.43
YOLOv5s	0.892	0.717	0.818	**526.32**	**16.4**
YOLOv8s	0.884	0.686	0.779	500	28.4
YOLOv10s	0.875	0.721	0.805	285.71	24.4
YOLOv11s	0.888	0.703	0.792	**526.32**	21.3
FasterRCNN	0.528	0.739	0.753	26.49	470.86
**PSD-YOLO (Ours)**	0.895	**0.744**	**0.825**	232.56	24.2

## Data Availability

The data that support the findings of this study are available from the corresponding author upon reasonable request.
